# Assessing the Driver’s Current Level of Working Memory Load with High Density Functional Near-infrared Spectroscopy: A Realistic Driving Simulator Study

**DOI:** 10.3389/fnhum.2017.00167

**Published:** 2017-04-05

**Authors:** Anirudh Unni, Klas Ihme, Meike Jipp, Jochem W. Rieger

**Affiliations:** ^1^Department of Psychology, University of Oldenburg Oldenburg, Germany; ^2^Institute of Transportation Systems, German Aerospace Research Center Braunschweig, Germany

**Keywords:** working memory load, realistic driving scenario, n-back, fNIRS, multivariate prediction

## Abstract

Cognitive overload or underload results in a decrease in human performance which may result in fatal incidents while driving. We envision that driver assistive systems which adapt their functionality to the driver’s cognitive state could be a promising approach to reduce road accidents due to human errors. This research attempts to predict variations of cognitive working memory load levels in a natural driving scenario with multiple parallel tasks and to reveal predictive brain areas. We used a modified version of the n-back task to induce five different working memory load levels (from 0-back up to 4-back) forcing the participants to continuously update, memorize, and recall the previous ‘n’ speed sequences and adjust their speed accordingly while they drove for approximately 60 min on a highway with concurrent traffic in a virtual reality driving simulator. We measured brain activation using multichannel whole head, high density functional near-infrared spectroscopy (fNIRS) and predicted working memory load level from the fNIRS data by combining multivariate lasso regression and cross-validation. This allowed us to predict variations in working memory load in a continuous time-resolved manner with mean Pearson correlations between induced and predicted working memory load over 15 participants of 0.61 [standard error (SE) 0.04] and a maximum of 0.8. Restricting the analysis to prefrontal sensors placed over the forehead reduced the mean correlation to 0.38 (SE 0.04), indicating additional information gained through whole head coverage. Moreover, working memory load predictions derived from peripheral heart rate parameters achieved much lower correlations (mean 0.21, SE 0.1). Importantly, whole head fNIRS sampling revealed increasing brain activation in bilateral inferior frontal and bilateral temporo-occipital brain areas with increasing working memory load levels suggesting that these areas are specifically involved in workload-related processing.

## Introduction

Operating a car imposes high cognitive demands on the driver because information from the traffic signs, in-vehicle displays, and other traffic participants has to be integrated into a coherent situation representation. This representation needs to be dynamically updated with new information thus being especially challenging for the driver’s working memory (De Waard, 1996; da Silva et al., 2014). This means that the current level of workload of the driver is largely defined by the current working memory demands. Therefore, being able to reliably measure the momentary working memory load of the driver would be a major step into the direction of designing automation systems that are adaptive to the driver’s cognitive workload. Parasuraman and colleagues (Parasuraman, 1987; Byrne and Parasuraman, 1996; Kaber et al., 2001) coined the term ‘adaptive automation’ for systems that aim to adapt the level of automation and support of assistance functions to the current state of the operator and thus to keep him or her at an optimal level of engagement and cognitive workload. This idea is very elegant on the conceptual level and its potential has been shown in several lab studies (Kaber and Riley, 1999; Parasuraman et al., 2009; Prinzel et al., 2016). However, up to the present day, reliably detecting different levels of cognitive workload is challenging especially in realistic scenarios such as driving.

Human working memory is a limited capacity system that can hold a limited number of information chunks at a time (Miller, 1956; Baddeley, 2003). This number varies between individuals and tasks. In the field of cognitive psychology and neuroscience, the well-accepted n-back task is used as a benchmark to experimentally manipulate working memory levels (Kirchner, 1958). It is thought that there is a monotonous increase of working memory load with increasing n, the number of items to be memorized, until the individual working memory capacity is reached. Increased working memory load comes along with increased error rates and increases in physiological arousal and changes of the sympathetic influence on the heart (Backs and Seljos, 1994; Gramann and Schandry, 2009). Several studies attempted to assess changes in working memory load in realistic situations from peripheral physiological parameters such as heart rate, heart rate variability or skin conductance level (Solovey et al., 2014; Gable et al., 2015). However, relying on peripheral physiology has the disadvantage that changes in arousal are not specific to working memory load, but are also integral to emotions such as anger or joy (Sander et al., 2005) and related to physical activity or fatigue (De Waard, 1996). In order to disentangle different types of workload (Wortelen et al., 2016) from emotional states, and to obtain more specific assessments of cognitive states, multidimensional brain activation measurements could be a promising complement.

Neuroimaging studies suggest that the pre-frontal brain areas are involved in processes necessary for working memory (D’Esposito et al., 1999; Spitzer et al., 2014). A promising approach to assess working memory load in realistic environments is to measure brain activation of the operator with functional near-infrared spectroscopy (fNIRS). “FNIRS utilizes light in the infrared range to measure brain activity via relative hemoglobin concentration changes (Villringer et al., 1993).”

There have been a few previous studies where both fNIRS and electroencephalography (EEG) brain activation measurements have been used to assess and discriminate working memory load levels in humans. Sassoroli et al. (2008) classified three different working memory load levels in a simple lab setting using two channels placed on the left and right side of the forehead. Hirshfield et al. (2009) combined EEG and fNIRS modalities to explore user’s mental workload. They elicited three different workload levels by increasing the number of objects to be tracked and classified these workload levels independently for fNIRS and EEG and report higher classification accuracies on fNIRS data compared to EEG data. Frey et al. (2016) provided a framework for EEG-based evaluation of user experience by continuously estimating mental workload, attention and interaction errors in a controlled virtual environment.

Some studies in the aviation domain have used fNIRS to measure working memory load levels in air traffic control (ATC) operators, unmanned vehicle operators and pilots. For instance, Ayaz et al. (2012) used the sixteen optodes fNIR 100 system over the forehead and found effects of type of communication and three different levels of working memory load on prefrontal oxygenated-hemoglobin (HbO) levels in ATC operators in a flight simulator. Gateau et al. (2015) used the sixteen fNIRS-optodes configuration with coverage over the pre-frontal areas to implement an online fNIRS-based system that discriminated two levels of a pilot’s instantaneous working memory load in a flight simulator. Harrison et al. (2014) reported increasing mean oxygenation levels in a single channel placed over left pre-frontal cortex in ATC operators managing increasing air traffic density.

In the driving domain, both EEG and fNIRS have been used to measure the workload levels of drivers. Lei and Roetting (2011) estimated driver workload from EEG spectrum modulation while participants performed a combination of lane-change and a working memory tasks in a driving simulator. They found an 8–12 Hz (alpha) oscillatory power decrease and a 4–8 Hz (theta) power increase with increasing workload. Tsunashima and Yanagisawa (2009) compared fNIRS data from the pre-frontal areas while participants drove in a driving simulator with and without adaptive cruise control (ACC) and reported that participants showed less frontal lobe activations while driving with ACC. Yoshino et al. (2013) used fNIRS during driving on an actual expressway and reported significant changes in HbO levels around the frontal eye field during speed changes as compared to driving with constant speed.

Most of the workload-related studies were able to discriminate only two or three levels of working memory load in realistic scenarios. For adaptive automation, however, continuous quantification of working memory load is desired. Moreover, due to the limited spatial sampling, these studies provided limited information about other than pre-frontal brain areas predictive of working memory load in realistic tasks.

The current study uses whole head, high density fNIRS in combination with a modified version of the n-back working memory task to continuously quantify the current level of working memory load in a realistic driving task with varying demands and traffic conditions. We measured brain activation using 78-channel fNIRS throughout the entire driving time and used multivariate analyses approaches to predict the current working memory load level on a continuous scale. The close to whole head fNIRS sampling that we employed should allow us to extend current knowledge of brain motives predictive of working memory obtained in fNIRS studies which focused solely on pre-frontal activation patterns. We also recorded task-related behavior, driving behavior, and peripheral physiology to simultaneously assess working memory load with measures other than fNIRS recordings. Our hypothesis is that neural activation patterns in the pre-frontal and parietal areas will be the relevant features for estimating the level of working memory load since previous laboratory studies have shown that these areas are known to be involved in working memory-related processing (LaBar et al., 1999; Wagner et al., 2005; Postle, 2006; Koenigs et al., 2009; Salazar et al., 2012; Lara and Wallis, 2015; Ekman et al., 2016).

## Materials and Methods

### Design

While driving in the driving simulator, participants accomplished a speed regulation version of the digit-span/n-back tasks that was integrated into the driving task in order to induce working memory load. The conducted version of the task had five difficulty levels (*n* = 0, 1, 2, 3, 4).

### Participants

Nineteen volunteers (17 males) aged 19–32 years (Mean ±*SD* = 25.2 ± 3.7) participated in this study. All participants possessed a valid German driving license and gave written informed consent to participate prior to the experiment in accordance with the Declaration of Helsinki. The experimental procedure was in line with the guidelines of the German Aerospace Research Center and approved by the Ethics Committee of the Carl von Ossietzky University, Oldenburg. Participants received a financial reimbursement of 10 per hour.

### Experimental Set-up

**FIGURE 1:**
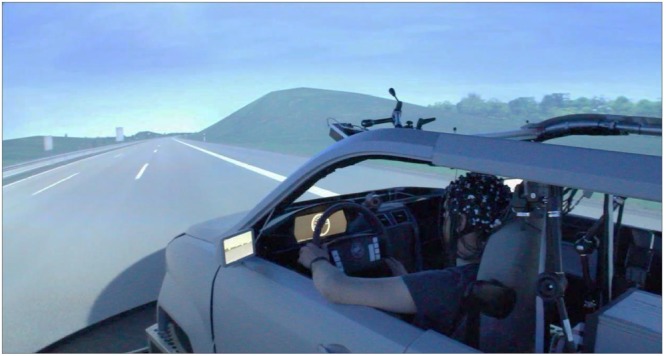
**Virtual Reality lab driving simulator at German Aerospace Center – photograph of experimental setup.** The participant is driving along the highway in this experiment. The fNIRS system and the computer are positioned in the backseat of the car behind the participant (not seen in this photograph).

The experiment was implemented in the virtual reality (VR) lab (**Figure 1**) with 360° full view at the German Aerospace Research Center (Fischer et al., 2014). Participants sat in a realistic vehicle mock-up and controlled the mock-up car in the driving simulation (Virtual Test Drive, Vires Simulationstechnologie, Bad Aibling, Germany) via a standard interface with throttle, brake pedal, steering wheel, and indicators. The track was a round course (64 km in total) consisting of a slightly curvy 3-lane-highway with standard lane widths. The simulation included concurrent traffic with density that varied over time.

The simulator equipment provided access to driving-related variables (movements of steering wheel, throttle and brake pedal as well as lane keeping behavior). Participants’ electrocardiogram (ECG) was recorded with a wearable physiological measurement system (HealthLab by SpaceBit, Eberswalde, Germany). Behavioral (task- and driving-related) and ECG data were used to assess the potential influence of n-back level on the driving behavior and peripheral physiology.

**FIGURE 2:**
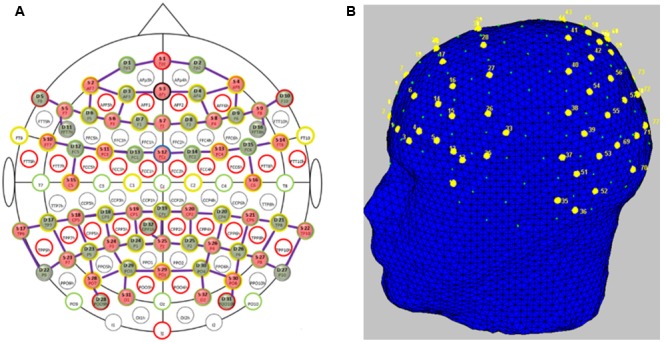
**Functional near-infrared spectroscopy probe placement. (A)** Topologic layout of the emitters (red), detectors (green) and the fNIRS channels (purple) on a standard 10–20 EEG system. Figure reproduced from NIRStar software with permission from NIRx Medical Technologies, LLC, USA. **(B)** FNIRS channels (labeled in yellow) superimposed onto the head model depicting coverage over the frontal and parietal and temporo-occipital areas. Figure generated using nirsLAB toolbox^1^.

Participants’ brain activity was measured using fNIRS. “FNIRS uses low-energy optical radiation in the near infrared range to measure absorption changes in the sub-surface tissues and obtain local concentration changes of oxy-hemoglobin (HbO) and deoxy-hemoglobin (HbR) as correlates of functional brain activity using modified Beer-Lambert law (Villringer et al., 1993; Sassaroli and Fantini, 2004).” We used two NIRScout (NIRX Medical Technologies) systems in tandem to acquire fNIRS signals. The system uses two wavelengths of 760 and 850 nm and outputs relative concentration changes of HbO and HbR. Thirty-two optical emitters and detectors each were used to obtain full coverage of the frontal, parietal and temporo-occipital cortices. In order to avoid crosstalk between the two systems, we arranged the optodes such that one system covered the frontal areas and the other system covered parietal and temporo-occipital areas. This arrangement left a gap approximately along the somatomotor cortices to separate the systems. We had 78 channels (combinations of emitters and receivers) in total (**Figure 2A**) for measuring HbO and HbR over nearly the whole head (**Figure 2B**). The average distance between an emitter and detector was approximately 3.5 cm. The sampling frequency of the tandem system was 1.955Hz.

### Experimental Paradigm

The working memory load manipulation was integrated into the driving via a speed regulation task. This manipulation was achieved using a combination of a digit-span and the n-back tasks to induce five different n-back levels (*n* = 0, 1, 2, 3, 4). In a classical n-back task, a stream of stimuli, e.g., numbers are presented. The participants need to compare the currently presented number with the number that occurred n steps back and provide a response (e.g., button press, mouse click) if both numbers are the same. In our modified n-back task, the participants had to continuously update, memorize and recall the previous n numbers, which were represented as speed signs and adjust their speed to the speed sign that occurred n steps back. The speed signs were distributed such that participants passed a new speed sign roughly every 20 s (i.e., the length of each trial).

**FIGURE 3:**
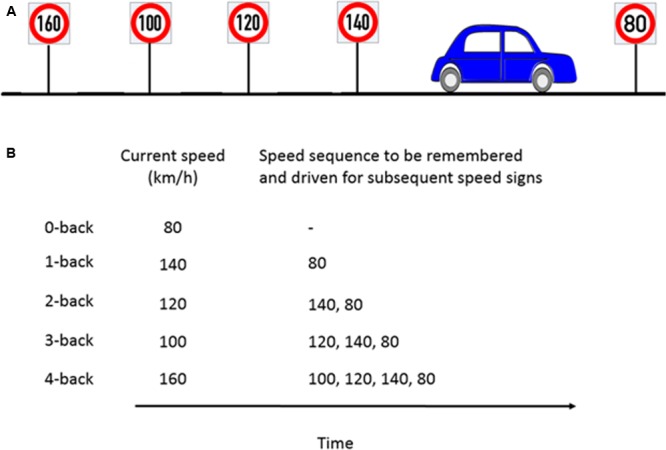
**Example of n-back experimental paradigm to manipulate cognitive workload. (A)** Consider a scenario where the participant is about to pass the 80km/h speed sign and the previous four speed signs were as shown in the schematic. **(B)** For the corresponding n-back task, participants had to memorize the last n speed signs and drive at the nth speed sign which occurred previously.

We first performed a training session where the participants drove the driving simulator over each of the five different n-back levels twice. The training session lasted for 20 min. In the experimental session, participants then drove with ten speed signs for each n-back level. Each new speed sign was considered as a single trial. Each n-back level was repeated twice, thus resulting in 100 trials in total (2 repetitions × 5 n-back levels × 10 trials) which lasted approximately 30 min and had a pseudorandomized order of the n-back levels with the constraint that the same n-back level was never driven twice in a row. The order of the n-back levels was balanced across participants. In addition, the speed signs varied from 60 to 140 km/h (in steps of 10 km/h) and presented in random order to avoid sequencing effects. At the beginning of a new n-back condition, a message was displayed for 5 s on the VR-screen to inform the participant about the n-back level to accomplish next. The example in **Figure 3A** illustrates the nature of the task. In this example, the participant is about to pass the 80 km/h speed sign and the previous four speed signs were 140, 120, 100, and 160 km/h, respectively. **Figure 3B** shows the speed that the participant is supposed to drive and the speed sequence to be remembered for the corresponding n-back task.

For a 0-back task, as soon as participant encounters the 80 km/h speed sign, (s)he has to adjust and maintain the speed close to 80 km/h. For a 1-back task, the participant had to drive at the speed indicated by the previous speed sign (hence the name 1-back), here 140 km/h, and remember 80 km/h which (s)he would drive at the next speed sign. For a 4-back task, the participant had to drive at the speed that occurred four speed signs previously, here 160 km/h, and remember the four element speed sequence, e.g., 100, 120, 140, and 80 km/h. This way, the participants had to update, memorize and recall a sequence of n speed signs and adjust their speed accordingly. Participants were allowed to take 3 s before and after passing a new speed sign to adjust their speed to the correct speed which was ±5 km/h around the target speed. After this interval, they were prompted by a visual message on the screen to stay in the correct speed range each time they drove more than 5 km/h above or below the target speed. The message remained on the screen as long as the participant drove outside the requested speed range. However, using this message to find the correct speed instead of remembering it would have led to very low performance as different speed signs appeared approximately every 20 s and we used 9 different target speeds which the participant was never explicitly told. The 17 s remaining after the warning message first appeared was too short to systematically scan the potential range of speeds and we found no indication of such a strategy in any of our participants. Because the task was performed with concurrent traffic, participants also had to consider the current traffic situation throughout the task which included lane changes.

For any n-back task, the participant needs to first pass through n successive speed signs before regulating the speed. For this reason, when a new n-back task begins, the participant was instructed to drive at the speed shown by the first speed sign.

### Data Analysis

#### Behavioral and Peripheral Physiological Parameters

We employed linear mixed effects analysis (West et al., 2014) to test linear relationships between the n-back condition (fixed effect), and driving behavior or physiological parameters, respectively. We entered intercepts for participants, trial number and target speed (i.e., the speed supposed to be driven in that trial) as random effects in the model. *P*-values were obtained by likelihood ratio tests (χ^2^-tests) of the full model with the effect in question against the model without the effect in question (West et al., 2014). We report the slope of the fixed effect of the model (n-back level) for each of the respective dependent variable as an unstandardized effect size and r as a standardized effect size. For the calculation of r, we used the Satterthwaite approximation [R package lmerTest (Bates et al., 2015)] to obtain the degrees of freedom (df) and *t*-values and the well-known relation *r* = sqrt(*t*^2^/(*t*^2^+df).

The driving behavior parameters included the proportion of time the participants drove in the correct speed range, the reaction time for the speed adjustments, brake, throttle and steering variance and the average deviation from the lane center (phases before and after lane change were omitted for determining the deviation from the lane center (34% of the data samples)]. The proportion of time in the correct speed range was the time during which participants drove at the target speed (±5 km/h tolerance) in relation to the total time for that trial (excluding the transition time of 3 s after the speed sign). The reaction time was calculated as the time that participants needed to reach the target speed (±5 km/h tolerance). It was measured from the moment when they passed the speed sign, with the constraint that they continue to drive at the target speed during the course of the trial. Reaction time was only calculated on correct trials.

For the driving and the n-back-task-related parameters, data right before and after passing the speed sign (±3 s) were not taken into account. Trials with extreme values (i.e., more than three standard deviations from the mean per participant) were removed from analysis. For the analysis of the correctness of the speed range, the reaction time, and the average deviation from the lane center, we removed less than 1% of the trials using this criterion. For throttle and steering variance, we removed less than 2% of the trials. For the analysis of brake variance, 12.4% of the trials were eliminated.

We calculated the heart rate and the Root Mean Square of Successive Differences (RMSSD) as a measure of heart rate variability from the ECG data to assess potential influence of n-back level on peripheral physiology. Heart rate and RMSSD were calculated per trial (i.e., over a time period of roughly 20 s). Trials with extreme values (i.e., more than three standard deviations away from the mean per participant) were removed from analysis. With this criterion, we removed 10.1% of the data for the heart rate and less than 1% for the RMSSD data.

#### Working Memory Capacity

In order to assess potential differences in the working memory capacities between participants, we used the Working Memory Capacity (WMC) test battery developed by Lewandowsky et al. (2010) to calculate scores for each participant. The test battery consists of four working memory tasks viz. sentence-span task, operation-span task, spatial short term memory task and memory updating task. We performed only the memory updating task as it closely resembles the working memory load manipulation induced by our experimental paradigm. The memory updating task was to remember an initial set of digits which was presented in a separate frame on the screen, and to continuously update these digits through simple arithmetic operations. Each correct trial was awarded 1 point. On average, our participants scored 38.4 [standard deviation (STD) 10.7] points out of 60 possible points. One participant scored less than two standard deviations than the mean score and was excluded from further analysis.

#### FNIRS Data Processing

**FIGURE 4:**
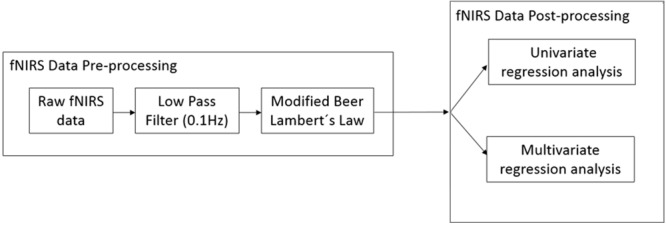
**Overview of the analysis pipeline for fNIRS data**.

**Figure 4** provides an overview of the fNIRS data analysis pipeline.

#### FNIRS Data Pre-processing

The raw data recorded from fNIRS is not just influenced by brain activity but also due to other systemic physiological artifacts (cardiac artifacts, respiration rate, Meyer waves) and movement artifacts causing the signal to be noisy. An overview of the fNIRS artifacts can be found in Cooper et al. (2012). The raw data was pre-processed using the nirsLAB analysis package (Xu et al., 2014). We use a low-pass filter (finite impulse response with least-square error minimization) with a cut-off frequency of 0.1 Hz to reduce these artifacts. We used the Gratzer Spectrum to obtain the molar extinction coefficients of HbO and HbR corresponding to wavelengths of 760 and 850 nm, respectively (Prahl, 1999). The corresponding molar extinction coefficients are 𝜖_760_ = [1486.59 3843.71] and 𝜖_850_ = [2526.39 1798.64] M^-1∗^cm^-1^ (nirsLAB, NIRx Medical Technologies). The differential path length factor takes into account the increased distance the light path travels from the emitter to the detector because of scattering and absorption effects. The differential path length factors for HbO and HbR were 7.25 and 6.38, respectively (Essenpreis et al., 1993). We then applied the modified Beer Lambert’s law to convert the data from voltage (μV) to relative concentration change (mmol/l) (Sassaroli and Fantini, 2004). For the Beer-Lambert law calculation, the source-detector distance for all channels was less than 4 cm. The exact source-detector distance for each NIRS channel was computed by nirsLAB according to the corresponding distances between emitter and detector pairs on the NIRS cap. nirsLAB has a built-in function to measure the signal-to-noise ratio (SNR) for each NIRS channel by calculating the coefficient of variation (CV) for unfiltered raw data (Schmitz et al., 2005; Schneider et al., 2011):

$$\begin{array}{c} \mathrm{CV}\;=\;\left(\frac{\;\sigma }{\;\mu }\right)\;*\;100\% \end{array}$$

Here, σ and μ are the standard deviation and the mean of the data of each NIRS channel over the entire duration of the experiment. Channels with a CV greater than 20% were rejected from further analysis. On average, 64 channels were retained per participant (STD 7).

The trials were extracted based on the time-stamps associated with the labels corresponding to each n-back condition. During the analysis, we removed trials where the participants couldn’t achieve the target speed (∼8% over all participants). This was done because for trials with errors, we weren’t sure if the participant was continuing to focus on the task or if he was unable to cope up with the cognitive demands of the task and had already given up at an earlier stage.

#### FNIRS Data Post-processing

##### Principal component analysis

We used the method of Principal Component Analysis (PCA) on the pre-processed fNIRS data in order to reduce the amount of noise in the data to increase the SNR. This method performed an orthogonal transformation of the fNIRS data into a set of linearly uncorrelated variables, i.e., ‘principal components’ (PCs) such that the first principal component (PC) accounted for the largest variance in the data, and each successive PC explained the largest possible variance in the data but was orthogonal to the preceding PC. If we assume noise to have a Gaussian distribution, then each of the PCs will also contain identically distributed Gaussian noise. However, since most of the total variance will be concentrated in the first few PCs compared to the same noise variance, the first few PCs achieve a higher SNR. The latter PCs will mostly be dominated by noise and these components were removed from the analysis before transforming the PCs back into the original space, i.e., the time-series fNIRS data.

The instantaneous time-series fNIRS HbR data was first split into test and training data. The selection of PCs for each participant was carried out in cross-validation loop nested into the training phase of the cross-validation loop used to test generalization of the multivariate lasso regression model (see next section). On average, the first 25 PCs were selected over 15 participants (STD 11).

##### Multivariate cross-validated prediction of working memory load level

We used multivariate lasso regression (Hastie et al., 2009) implemented in the Glmnet toolbox^2^ to find channel-wise weights for instantaneous fNIRS HbR data to predict the working memory load level.

The λ parameter which determines the overall intensity of regularization was optimized internally by Glmnet in the training phase of the cross-validation of the lasso regression model.

We used a standard nested cross validation procedure (Hastie et al., 2009) to train the model and test generalization performance. Each loop implemented a 10-fold cross-validation. The outer cross-validation loop tested the generalization of the regression model with the optimized hyperparameters (number of PCs and λ). The hyperparameters were optimized in an inner cross-validation loop which was implemented in the training phase. Cross-validation avoids overfitting of the data to the model and provides an estimate of how well a decoding approach would predict new data in an online analysis (Reichert et al., 2014).

We used the same method to estimate the predictions of working memory load levels from the trial-wise heart rate and RMSSD values.

##### Univariate correlation analysis

Multivariate decoding models can be hard to interpret regarding the specific brain areas which are sensitive to working memory load-related changes (Reichert et al., 2014; Weichwald et al., 2015). In order to simplify the interpretation of brain activation measurements, we regressed the HbR fNIRS measurements on the current n-back working memory load levels for each channel separately and calculated univariate Pearson’s correlations (*r*_uvr_) from the regression for each participant. Pearson’s correlations quantify the strength of the relation between induced working memory load and measured brain activation.

For generalization of the individual correlation maps to our population sample, we calculated weighted average correlation maps across all participants (*r*_avg_). Therefore, the single-subject Pearson’s correlations (*r*_uvr_) for each fNIRS channel were weighted with the participant’s Pearson’s correlation (*r*_mvr_) from the multivariate regression analysis:

$$\begin{array}{c} r_{\mathrm{avg}}\left(i\right)=\frac{\sum_{i,n\;=\;1}^{i,n}r_{\mathrm{uvr}}(i)\;r_{\mathrm{mvr}}(n)}{\sum_{1}^{n}r_{\mathrm{mvr}}(n)} \end{array}$$

In the above equation, *n* is the total number of participants and *i* is the total number of fNIRS channels.

## Results

### Participants

Data from three participants were excluded due to a large number (>50%) of noisy channels in the fNIRS and one because of low performance in the WMC test. Thus, data from fifteen participants are reported in the following fNIRS analyses. Due to failure of the data acquisition of the behavioral and the heart rate parameters, three additional participants had to be excluded from the analyses of behavioral and peripheral physiological data.

### Behavioral and Peripheral Physiological Results

**Table 1** lists the mean values and standard deviations of the behavioral and heart rate parameters for the twelve participants included in these analyses.

**Table 1:** Descriptive statistics (mean values and standard deviation) of the task-related, driving behavior, and physiological parameters in the five n-back conditions.

		0-back	1-back	2-back	3-back	4-back
Task-related	Time in correct range (in %)	92.3 (0.04)	86.0 (0.09)	75.8 (0.18)	69.9 (2.54)	71.0 (18.6)
	Reaction time (in seconds)	1.35 (0.61)	1.63 (0.65)	1.84 (0.69)	2.05 (1.26)	2.04 (1.16)
Driving behavior	Brake variance (in a.u.)	0.12 (0.14)	0.11 (0.14)	0.11 (0.63)	0.44 (0.59)	0.51 (0.68)
	Throttle variance (in a.u.)	0.26 (0.08)	0.30 (0.09)	0.28 (0.08)	0.24 (0.11)	0.23 (0.11)
	Steering variance (in 10^-4^ radians)	0.69 (0.10)	1.28 (0.16)	0.42 (0.09)	1.31 (0.19)	0.59 (0.18)
	Deviation from lane center (in meters)	0.15 (0.02)	0.19 (0.04)	0.15 (0.04)	0.18 (0.04)	0.16 (0.03)
Physiology	Heart rate (in bpm)	73.8 (12.2)	75.2 (12.3)	75.8 (12.7)	76.3 (13.4)	77.7 (13.6)
	RMSSD (in milliseconds)	39.5 (17.0)	38.2 (18.2)	36.1 (16.1)	35.5 (16.1)	35.2 (17.8)

*a.u. = arbitrary units.*

Considering driving behavior, we find significant effects of the n-back condition on the time participants drove at the correct speed [χ^2^ = 12.02, *p* < 0.001, approximated *r* = -0.75, decrease per n-back level (slope): 6.6%, SE = 1.5%], the reaction time (χ^2^ = 4.25, *p* < 0.05, *r* = 0.47, increase per n-back level: 0.23 s, SE = 0.10) and the brake variance (χ^2^ = 7.44, *p* < 0.01, *r* = 0.58, increase per n-back level: 0.08, SE = 0.04). The time during which the participants drove at the correct time decreased, while the time they needed to reach the correct speed and the variability of the brake pedal position increased with increasing working memory load. The n-back condition had no significant effect on throttle variance (χ^2^ = 2.90, *p* = 0.09, *r* = -0.21, decrease per n-back level: 0.01, SE = 0.01), steering variance (χ^2^ = 2.01, *p* = 0.16, *r* = -0.29, decrease per n-back level: 0.8^∗^10^-5^ radians, SE = 0.5^∗^10^-5^ radians) and lateral deviation (χ^2^ = 0.05, *p* < 0.83, *r* = -0.07, decrease per n-back level: 0.007 m, SE = 0.03 m). These results indicate that working memory load can have an effect on safety relevant driving behaviors.

Working memory load level had a significant effect on heart rate (χ^2^ = 9.22, *p* < 0.001, *r* = 0.64, increase per n-back level: 0.89 bpm, SE = 0.25 bpm) and heart rate variability (RMSSD: χ^2^ = 3.89, *p* < 0.05, *r* = -0.59, decrease per n-back level: 1.24 ms, SE = 0.57 ms). Heart rate increased and heart rate variability decreased with increasing n-back level.

In order to compare working memory load predictions between brain and peripheral physiology data, we predicted the n-back levels from the trial-wise data for heart rate and heart rate variability using the lasso regression model on a two dimensional feature space. The mean Pearson’s correlation was 0.21 (SE = 0.1) across all participants. Note that unlike the LMM, the lasso prediction is 10-fold cross validated (i.e., it tests generalization to new data) and does not include random effects.

### FNIRS Results

The fNIRS results of this study are focused primarily on the HbR signal as some studies have shown that HbR signals are less influenced by systemic physiological artifacts like cardiac pulsation, respiration or Mayer wave fluctuations (Obrig et al., 2000; Zhang et al., 2005, 2009; Huppert et al., 2009). Some studies have also reported that HbR tends to show higher correlation with the BOLD response when compared to HbO (MacIntosh et al., 2003; Huppert et al., 2006; Schroeter et al., 2006; Foy et al., 2016).

### Multivariate Prediction of Working Memory Load

**FIGURE 5:**
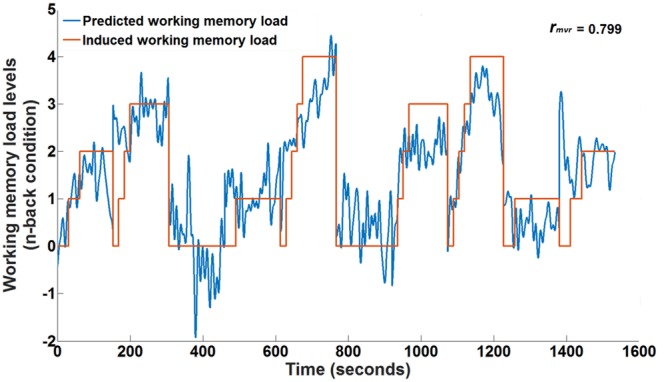
**Ten-fold cross-validated prediction of working memory load from HbR fNIRS measurements using multivariate regression analysis for an example participant (participant 3) over one session**.

**Figure 5** depicts the time course of working memory load induced by the n-back task (orange curve) together with the working memory load predicted by the fNIRS multivariate regression model (blue curve) for participant 3. This participant showed the highest correlation between the predicted and induced working memory load level. The Pearson’s correlation between the two curves is almost 0.8. It can be seen from **Figure 5** that the predicted working memory load nicely follows the induced working memory load and could be used to predict variations in cognitive working memory load levels. There are intervals when the model seems to over- or under-estimate the working memory load level. This may be due to the incomplete model which currently neglects the workload imposed by the concurrent driving task in the changing traffic situations. In spite of this, we are able to achieve satisfactory predictions. The mean Pearson’s correlation and the standard error across all participants were 0.61 and 0.04, respectively. **Table 2** lists the individual Pearson’s correlations (*r*_mvr_) for the fifteen participants for the whole head coverage. All multivariate correlations were determined in a 10-fold cross-validation to evaluate generalization to new data the model had not ‘seen’ before to approximate an online analysis.

**Table 2:** Multivariate Pearson’s correlations (*r*_mvr_) obtained from all participants after 10-fold cross-validated working memory load prediction from HbR fNIRS data using multivariate regression (*p* < 0.01 for all participants).

Participant number	P1	P2	P3	P4	P5	P6	P9	P10	P11	P13	P14	P15	P16	P17	P19
*r*_mvr_	0.7	0.69	0.8	0.54	0.58	0.32	0.54	0.72	0.31	0.57	0.61	0.77	0.75	0.72	0.59

To demonstrate the advantage of whole head fNIRS recordings over recordings restricted to the forehead (Ayaz et al., 2012; Harrison et al., 2014; Gateau et al., 2015), we re-ran our analysis using only the 12 frontal channels over the forehead region. The spatial distribution of these channels extended from locations F5 up to F6 laterally and two rows inferior toward the nasion [AF7 up to AF8 including AFz and Fpz (see **Figure 2A**)] corresponding to the International 10–20 system. In this case, the mean Pearson’s correlation over all participants drops to 0.38 (SE 0.04). A Wilcoxon Signed-Rank test indicated that Pearson’s correlations obtained from whole head measurements were significantly higher compared to only the frontal channels (*Z* = 3.39, *p* < 0.001) indicating that recordings restricted to the forehead are likely to miss informative brain activation.

### Localization of Predictive Brain Areas

**FIGURE 6:**
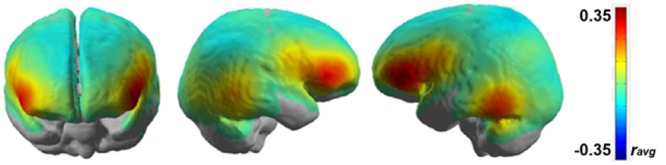
**Mean correlation map showing working memory load related brain areas from HbR-fNIRS.** Positive Pearson correlations indicate linear increase in brain activity with increasing working memory load. Data are overlaid on a standard template brain.

The next analysis was to characterize the pattern of brain areas that conveys information about the current level of working memory load. As the multivariate fNIRS regression model linearly combines multiple channels to predict current working memory load, one approach would be to interpret the weights of these decoding models. However, decoding model weights are hard to interpret for various reasons (Reichert et al., 2014; Weichwald et al., 2015). Therefore, we performed a univariate regression analyses separately for each channel. Univariate single channel analysis typically does not reach the predictive power of the full multivariate model but it can reveal how much predictive power a channel can in principle contribute to the full model. **Figure 6** depicts the average weighted correlation maps obtained from univariate HbR fNIRS regressions.

The results from this group level analysis showed that highest correlations of brain activation for working memory load are found bilaterally in the inferior frontal areas (*r*_avg_∼0.3), potentially reflecting activation changes in the ventral and dorsal pre-frontal cortices which have been previously implicated in working memory (Courtney, 2004; D’Esposito and Postle, 2015). Additional informative channels can be seen bilaterally in the temporo-occipital areas although the average linear trend is not as strong there as it is in the frontal areas.

**FIGURE 7:**
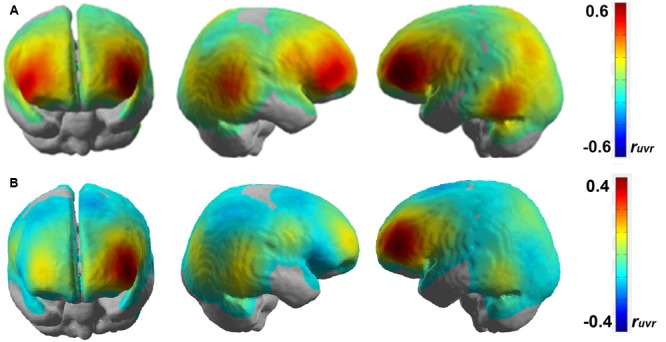
**Correlation map obtained by regressing HbR for each fNIRS channel over n-back working memory load level for (A)** participant 3 and **(B)** participant 4 on a standard brain template.

While the group level analysis is good for generalization across participants, it might not be ideal for identifying neural networks due to the averaging of the brain activations on a channel level across participants. For this, it is necessary to analyze the results on a single-subject level. **Figure 7** shows the correlation map representing working memory load-related brain areas after performing a channel-wise linear regression of HbR fNIRS data over the n-back tasks for two participants (participants 3 and 4).

The brain activation patterns shown in **Figure 7** closely resemble that of the group level analysis as shown in **Figure 6**. It can be seen that brain activation covaries with working memory load in the bilateral inferior frontal areas and the bilateral temporo-occipital areas. Additionally, we also observe some predictive activation in the inferior parietal areas. A correlation coefficient of *r*_uvr_∼0.4–0.6 in the left lateral frontal areas is statistically significant (*p* < 0.05) and shows a strong correlation between working memory load and brain activation level.

## Discussion

The aim of this research was to investigate if fNIRS-based brain activation measurements could be used to predict variations in cognitive workload levels in realistic driving simulation. To our knowledge, this study is the first to measure whole head, high density fNIRS in a driving simulator to characterize brain motives predictive of working memory load over a wide range of task complexities from very easy to overload. Our results indicate that whole head fNIRS in combination with multivariate regression can continuously predict momentary working memory load, across multiple levels although driving demands varied independently. We find that the bilateral inferior frontal areas and the bilateral temporo-occipital areas predict working memory load level in our n-back speed adjustment task. Other physiological and behavioral measures produced more complex prediction patterns, if any.

Using fNIRS HbR brain activation as the main physiological measurement of workload, we demonstrate that it is possible to continuously predict variations in working memory load over five different levels of an n-back task in a naturalistic driving scenario where participants performed multiple concurrent tasks over a long period of time. Our approach of using multivariate lasso regression in combination with cross-validation was tailored to explore the usefulness of multichannel whole head fNIRS for time-resolved characterization of workload levels. Multivariate modeling allowed us to predict working memory load variations in a continuous time-resolved fashion with relatively high accuracy. The cross-validation approach allowed us to estimate generalization of the regression model to new data which would be necessary in online tracking (Reichert et al., 2014), e.g., for adaptive assistive systems.

Most of the previous fNIRS related workload studies in the transportation domain (Kojima et al., 2005; Tomioka et al., 2009; Ayaz et al., 2012; Gateau et al., 2015; Foy et al., 2016; Sibi et al., 2016) focused only on frontal brain activations, primarily due to the ease in preparing the optodes around the forehead. On comparing the multivariate predictions from the whole head measurements with only the 12 frontal channels around the forehead, the mean Pearson correlation dropped by 37.7%. These results were statistically significant indicating that whole head measurements capture additional predictive brain activation.

The close to whole head fNIRS sampling allowed us to characterize the brain areas predictive of working memory load level. Because weights of regularized multivariate decoding models can be hard to interpret (Reichert et al., 2014; Weichwald et al., 2015), we used univariate linear regression to determine for each channel separately if it can predict the current working memory load level. These analyses revealed multiple predictive brain areas. The group level analysis showed that the bilateral frontal and the bilateral temporo-occipital areas had the highest predictive power. In these areas, brain metabolism increased with increasing working memory load. The single-subject analysis revealed parietal activation patterns over only seven participants. This was also the reason why they did not show up in the group level analysis as they are lost due to averaging.

The pattern of predictive brain areas we found is in concordance with the notion that the bilateral inferior frontal areas, potentially including activation changes in the ventral and dorsal pre-frontal cortices (VLPFC and DLPFC) which have known to be involved in the maintenance, manipulation and coordination of information (Postle, 2006; Lara and Wallis, 2015). The DLPFC is thought to be a major anatomical correlate of the central executive that plays a key role in working memory processing (Baddeley, 2003). However, to confirm the exact localization in the brain, structural MRI scans of the optode positions need to be performed on the individual brain which was not possible during the course of our experiment. The bilateral temporo-occipital areas might be more domain-specific which have been shown to be involved in lexico-semantic and visual working memory (Slotnick, 2010; Ardila et al., 2015; Barriga-Paulino et al., 2015). Importantly, our results indicate that whole head fNIRS is capable of revealing a functionally specific and spatially distributed motive of brain areas capable of predicting working memory load independent of multiple concurrently varying cognitive task demands. This specificity is necessary for assessing working memory load in real world conditions where operators are typically engaged in more than one task at a time.

Multiple behavioral and peripheral physiological parameters were, on average, significantly affected by the n-back level. We found that participants drove for a longer time at the incorrect speed and needed more time to reach the designated speed with increasing workload. Moreover, participants were more variable in their braking behavior, showed an elevated heart rate and lowered heart rate variability when the workload increased. These results are in agreement with previous studies (Zeitlin, 1995; Buld et al., 2014) who reported covariation of braking variance with increasing workload and with studies (Solovey et al., 2014; Gable et al., 2015) who reported that cognitive workload increases arousal resulting in varying sympathetic influence on the heart as reflected in heart rate increase and decreased heart rate variability. It should be noted that while the braking variance showed a statistically significant effect with varying n-back tasks, it is not a continuous measure. The braking behavior can easily vary depending on the external traffic situation and thus, cannot be used to reliably predict driver workload.

Although peripheral physiological measurements like heart rate or heart rate variability can be measured with relatively little effort, potentially continuously even during driving, these measures are relatively unspecific and may also occur due to changes in emotions or physical activity (e.g., fatigue) (De Waard, 1996; Sander et al., 2005). Moreover, low dimensional measures such as heart rate and its variability cannot resolve multidimensional cognitive processes. Spatially resolved brain activation measurements can be more specific to cognitive processes as they are recorded at the location where these processes are unfolding. Moreover, spatially resolved brain activation measurements are multidimensional which is necessary to discriminate and selectively assess loads on different types of cognitive and affective processes. However, future studies should test if peripheral physiological measurements can augment brain activation measurements for working memory load prediction.

Some studies report that HbO has a better SNR and is better correlated with the BOLD response as compared to HbR (Strangman et al., 2002; Yamamoto and Kato, 2002; Cui et al., 2011; Noah et al., 2015). In this study, the HbO signal showed some non-stationarities over the entire course of the experiment. A slow variable drift of the HbO signal was visible over a significant number of channels which hampered its analysis in the particular implementation of our experimental paradigm. Classic block paradigms are designed to include a period of rest before the presentation of stimuli so that there is sufficient time for the hemoglobin concentration changes to settle back to baseline levels. However, our paradigm was designed to mimic realistic slow and continuous workload variations and therefore each n-back level was immediately followed by the next. The HbR signal seemed less influenced by these non-stationarities and hence, the fNIRS results of this study focused only on the HbR signal.

Most fNIRS studies consider cardiac activations as a physiological artifact and try to reduce the influence of the cardiac activity on the fNIRS signal. However, a few studies have shown that heart rate and the heart rate variability can be used as an indicator of mental workload (Kamath and Fallen, 1993; Miyake, 2001; Hjortskov et al., 2004; Durantin et al., 2014). In our study, we tried to predict the working memory load levels from the trial-wise data for heart rate and heart rate variability using the lasso regression model. However, the prediction rates were clearly inferior to those obtained with both frontal only and whole head fNIRS data. In the future, we plan to combine data from both fNIRS and ECG data-streams and provide this as an input to the model. We expect that the integration of this data will provide additional independent information to the model which might enable us in achieving better prediction rates.

We expect that the results obtained in this study can be further improved by improving the analysis techniques. Here, we used a linear model for the predicting working memory load levels. However, Wortelen et al. (2016) implementing working memory modeling in the cognitive architecture for safety critical task simulation (CASCaS) (Lüdtke et al., 2009), and testing the model on the same paradigm as we have used here, predict a compressive non-linearity for increasing workload levels. One reason for this compression could be that working memory is capacity limited (Miller, 1956; Baddeley, 2003) and therefore increases in task induced working memory load will lead to smaller increases in working memory load levels at high workload levels. Hence, the HbR levels should saturate for workload induction levels that reach or exceed the capacity limit or may even invert when the participant disengages due to overload. Consequently, we concentrated our analysis here on correct trials to mitigate the effect of the expected dissociation between experimentally induced (i.e., expected) and actual workload level at high loads. Future studies may consider using regression models with a non-linear link function to further improve memory workload prediction. Moreover, our existing regression model is incomplete since it includes only the working memory load imposed by the n-back task but neglects the workload imposed by other concurrent driving tasks in the changing traffic situations. In the future, we plan to augment our existing model by integrating the influence of the traffic dynamics into the workload model to get much better prediction rates. Furthermore, Herff et al. (2014) integrated fNIRS data over different time windows and showed an increase in the prediction accuracy with increasing temporal integration windows. In our study, we only integrated information spatially over multiple channels to improve the workload predictions. We would expect our model to achieve much better predictions in the future by integrating information in both temporal and spatial domains.

The results of this study are of interest for the application in vehicles in an adaptive assistance or automation system. Future partial autonomous cars may well ask the human to drive the car in certain situations, for example when the automation is at its system limits or in order to prevent loss of skill for the driver. In the latter case, a real-time onboard driver workload monitoring system could help to time such take-over requests or adaptively remove the degree of support in phases of low workload. Although one may argue that the cognitive workload while driving may not be as high as the 4-back task in our experiment, there are many factors (e.g., road layout, traffic density, or speed) that influence task difficulty and impose load on the driver’s cognitive resources (Fuller, 2005; Fastenmeier and Gstalter, 2007). Thus, being able to identify differences from low to high cognitive workload will help to understand the momentary capability of the driver and to provide tailored assistance.

## Conclusion

Our study demonstrates that whole head fNIRS is a valuable neuroimaging modality to assess the driver’s cognitive workload level in realistic scenarios with concurrently executed tasks. Such an approach could pave the way for a driver assistance system that can adapt its current level of engagement or automation to the driver’s state and by that, increase the safety of highly automated vehicles.

## Author Contributions

JR, MJ, AU, and KI planned the research. Data collection was done by AU and KI. Data analysis was carried out by AU, KI, and JR. AU, KI, JR, an MJ prepared the manuscript. AU and KI contributed equally.

## Conflict of Interest Statement

The authors declare that the research was conducted in the absence of any commercial or financial relationships that could be construed as a potential conflict of interest.

## Footnotes

1 https://www.nitrc.org/projects/fnirs_downstate

2 http://web.stanford.edu/~hastie/glmnet_matlab/
