# Corrigendum: Assessing the Driver's Current Level of Working Memory Load With High Density Functional Near-infrared Spectroscopy: A Realistic Driving Simulator Study

**DOI:** 10.3389/fnhum.2018.00498

**Published:** 2018-12-18

**Authors:** Anirudh Unni, Klas Ihme, Meike Jipp, Jochem Rieger

**Affiliations:** ^1^Department of Psychology, University of Oldenburg, Oldenburg, Germany; ^2^Institute of Transportation Systems, German Aerospace Center, Braunschweig, Germany

**Keywords:** working memory load, realistic driving scenario, n-back, fNIRS, multivariate prediction

In the original article, there was an error. The analysis on participants' deviation from the lane center was incorrect. In the methods, we stated that “phases before and after lane change were omitted for determining the deviation from the lane center.” However, these phases were mistakenly included in the analysis and reported in the original paper.

A correction has been made to the **Data Analysis, Behavioral and Peripheral Physiological Parameters**, paragraph two:

“The driving behavior parameters included the proportion of time the participants drove in the correct speed range, the reaction time for the speed adjustments, brake, throttle and steering variance and the average deviation from the lane center (phases before and after lane change were omitted for determining the deviation from the lane center [34% of the data samples]). The proportion of time in the correct speed range was the time during which participants drove at the target speed (±5 km/h tolerance) in relation to the total time for that trial (excluding the transition time of 3 s after the speed sign). The reaction time was calculated as the time that participants needed to reach the target speed (±5 km/h tolerance). It was measured from the moment when they passed the speed sign, with the constraint that they continue to drive at the target speed during the course of the trial. Reaction time was only calculated on correct trials.”

A correction has also been made to the **Results**, **Behavioral and Peripheral Physiological Results**, paragraph two:

“Considering driving behavior, we find significant effects of the n-back condition on the time participants drove at the correct speed (χ^2^ = 12.02, *p* < 0.001, approximated *r* = −0.75, decrease per n-back level [slope]: 6.6%, SE = 1.5%), the reaction time (χ^2^ = 4.25, *p* < 0.05, *r* = 0.47, increase per n-back level: 0.23 s, SE = 0.10) and the brake variance (χ^2^ = 7.44, *p* < 0.01, *r* = 0.58, increase per n-back level: 0.08, SE = 0.04). The time during which the participants drove at the correct time decreased, while the time they needed to reach the correct speed and the variability of the brake pedal position increased with increasing working memory load. The n-back condition had no significant effect on throttle variance (χ^2^ = 2.90, *p* = 0.09, *r* = −0.21, decrease per n-back level: 0.01, SE = 0.01), steering variance (χ^2^ = 2.01, *p* = 0.16, *r* = −0.29, decrease per n-back level: 0.8^*^10^−5^ radians, SE = 0.5^*^10^−5^ radians) and lateral deviation (χ^2^ = 0.03, *p* = 0.87, *r* = −0.04, decrease per n-back level: 0.0005 m, SE = 0.003 m). These results indicate that working memory load can have an effect on safety relevant driving behaviors.”

In the original article, there was a mistake in Table 1, as published, due to the errors stated above. The corrected Table 1 appears above.

**Table 1:** Descriptive statistics (mean values and standard deviation) of the task-related, driving behavior, and physiological parameters in the five n-back conditions.

		**0-back**	**1-back**	**2-back**	**3-back**	**4-back**
Task-related	Time in correct range (in %)	92.3 (0.04)	86.0 (0.09)	75.8 (0.18)	69.9 (2.54)	71.0 (18.6)
	Reaction time (in seconds)	1.35 (0.61)	1.63 (0.65)	1.84 (0.69)	2.05 (1.26)	2.04 (1.16)
Driving behavior	Brake variance (in a.u.)	0.12 (0.14)	0.11 (0.14)	0.11 (0.63)	0.44 (0.59)	0.51 (0.68)
	Throttle variance (in a.u.)	0.26 (0.08)	0.30 (0.09)	0.28 (0.08)	0.24 (0.11)	0.23 (0.11)
	Steering variance (in 10^−4^ radians)	0.69 (0.10)	1.28 (0.16)	0.42 (0.09)	1.31 (0.19)	0.59 (0.18)
	Deviation from lane center (in meters)	0.15 (0.02)	0.19 (0.04)	0.15 (0.04)	0.18 (0.04)	0.16 (0.03)
Physiology	Heart rate (in bpm)	73.8 (12.2)	75.2 (12.3)	75.8 (12.7)	76.3 (13.4)	77.7 (13.6)
	RMSSD (in milliseconds)	39.5 (17.0)	38.2 (18.2)	36.1 (16.1)	35.5 (16.1)	35.2 (17.8)

*a.u. = arbitrary units*.

The authors apologize for these errors and state that this does not change the scientific conclusions of the article in any way. The original article has been updated.

## Conflict of Interest Statement

The authors declare that the research was conducted in the absence of any commercial or financial relationships that could be construed as a potential conflict of interest.

